# Racial and Socioeconomic Healthcare Disparities in Access to Chimeric Antigen Receptor T (CAR‐T) Cell Therapy for Blood Cancers

**DOI:** 10.1002/cam4.71457

**Published:** 2026-02-06

**Authors:** Hasini Warnakulasuriya, Ritika Tiwari

**Affiliations:** ^1^ MSc Public Health York St John University London UK; ^2^ Business and Health Studies York St John University London UK

**Keywords:** blood cancers, CAR‐T cell therapy, health disparities, health equity, minority groups

## Abstract

**Background:**

Health disparities remain a critical global public health challenge, particularly in access to advanced treatments for blood cancers. Racial and socioeconomic factors influence healthcare accessibility, contributing to inequities in patient outcomes. Despite the potential of CAR‐T therapy in treating blood cancers, disparities in financial resources, education, gender, and race hinder equitable access. This study evaluates literature on CAR‐T therapy to identify access disparities and proposes policy recommendations.

**Methods:**

The PRISMA‐ScR guidelines were followed for study selection and reporting. A comprehensive search strategy was used across databases like PubMed and Google Scholar, using keywords and MeSH terms. Inclusion criteria included peer‐reviewed studies in English since 2000. A basic quality appraisal was conducted to ensure the relevance and credibility of included studies, despite the diversity of study designs and the primary focus on mapping key themes across the literature.

**Results:**

Twenty‐five relevant (25) studies, including analytical studies, observational studies, and literature reviews, were analyzed. Findings indicate significant racial and socioeconomic disparities in CAR‐T therapy accessibility, with financial constraints, lack of awareness, and systemic biases limiting equitable distribution. Challenges include high treatment costs, lack of insurance coverage, and underrepresentation of minority groups in trials.

**Conclusion:**

Addressing these disparities requires targeted policy interventions, increased funding, and improved patient education. Continued research and collaboration are essential to ensure equitable access for all individuals.

## Graphical abstract

PRISMA flow diagram illustrating the study selection process for the scoping review, resulting in 25 studies included in the final review.

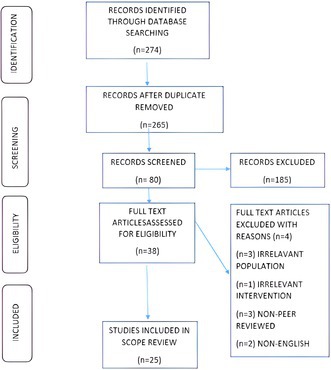

## 1 Introduction

### 1.1 Health Disparities

Health disparities are differences in health outcomes among particular groups. Differences in disease incidence, prevalence, mortality, and severity can be used to quantify these disparities [1].

In recent years, growing health disparities have become a global concern, particularly posing a huge threat to public health. Data suggests that social determinants of health such as the conditions in which people live, work, and learn can quickly change a society and impact the well‐being of society [1]. These determinants are the underlying cause of health disparities. The unequal distribution of resources, power, and wealth affects these variables, which could impact the disease incidence, access to care and mortality rate [2].

### 1.2 Socioeconomic Disparities

It is believed that socioeconomic disparities have a negative influence on the health outcomes of numerous illnesses according to the research [3] since these can prevent patients from receiving the full benefits of successful treatments as stated by the research [4] resulting in loss of functionality, physical and cognitive impairments, and even increased mortality rates. Public health is greatly impacted by racial and ethnic disparities since people from different ethnic backgrounds do not access healthcare services equitably [3]. Many studies have been conducted to determine the underlying causes of these disparities and create more equitable healthcare policies because of the wide discussion of these issues throughout history. Although there has been progress in narrowing these gaps, there is still much to be done to eliminate racial and ethnic disparities in healthcare [5]. Minority groups continue to receive lower‐quality healthcare than non‐minorities, despite the availability of advanced treatment options; this leads to worse health outcomes [6].

### 1.3 Financial Disparities

Financial disparities exacerbate health inequalities by limiting access to high‐quality healthcare [4]. Stressful living conditions and poor health outcomes are frequently caused by lower socioeconomic status. However, financial instability is not the only reason for some people to avoid medical care.

In addition to socioeconomic and racial disparities, health disparities can also stem from differences in gender, age, disability, and geographical factors [5].

### 1.4 Educational Disparities

While lower educational levels are linked to worse health outcomes, higher educational attainment is linked to improved health outcomes [7]. It also has an impact on behaviors like smoking and poor diet [8]. Disparities in education result in a lack of awareness of diseases, their symptoms, progression, and treatments. Health outcomes may deteriorate because of this ignorance, which is often aggravated by a lack of access to reliable information and social stigma. For instance, greater survival rates are associated with cancer awareness, but a lack of awareness lowers the possibility of a successful course of therapy [9]. Despite the effectiveness of CAR‐T cell therapy, many individuals cannot access it simply because they lack knowledge about the treatment.

### 1.5 Gender Disparities

Gender imbalance in healthcare is a critical concern, especially for women, who encounter more acute healthcare inequities. The undervaluation of women's health in society often results in delayed care and adverse health outcomes [10]. Particularly, women from underprivileged socioeconomic and racial backgrounds receive low‐quality treatment. These disparities can take many forms, including underrepresentation in studies, delayed diagnosis, and a disregard for women's health issues, which are frequently attributed to psychological reasons [10].

### 1.6 Blood Cancers and Treatment Methods

Hematologic malignancies which are also called blood cancers, such as leukemia, lymphoma, and multiple myeloma, originate from blood or lymphatic tissues and may be lethal [11]. With over 100 subtypes and different causes, blood malignancies rank fifth among all cancer forms in developed countries. Although intense treatment can cure some blood cancers, only 40% of them have the potential to be cured, and 60% of those can be fatal [12]. Early diagnosis is difficult due to the gradual onset of symptoms, which often appear as signs of other illnesses [12].

The course of treatment for blood cancers varies according to the type of cancer, patient age, metastasis, and progression. Surgery, chemotherapy, and radiotherapy are examples of conventional treatments; chemotherapy is frequently used as a first line of treatment. However, these therapies frequently have serious adverse effects, such as extended recovery periods and damage to healthy tissue [13].

### 1.7 Health Disparities and Blood Cancers

Health disparities include lower socioeconomic status, limited education, limited access to healthcare. Environmental factors are the main causes of cancer, which is also the top cause of death among Hispanics in the United States [14].

Approximately 60 million Hispanic people (18% of the US population) earn 30% less on average per year than non‐Hispanics. Hispanic children have the greatest incidence rates of leukemia (62.6 per million) compared to non‐Hispanic children (52.2 per million), making it the most prevalent blood cancer in this group [14]. Their five‐year survival rate is 90%, which is less than the 95% rate for white non‐Hispanic children. The prevalence of acute lymphoid leukemia (ALL) is higher among Hispanics than among other populations, and even with early detection, outcomes remain poorer [14]. Due to delayed care, lack of insurance, and restricted access to early treatments, Hispanics with Acute Promyelocytic Leukemia (APL), a curable form of acute myeloid leukemia, have a 5–10 year worse survival rate. Access to treatment is further restricted by the fact that 72% of Hispanics in the US do not have health insurance [14].

Black Americans have a 19% higher overall cancer rate than white Americans, making cancer the second most common cause of death for them [15]. Despite having the highest mortality and lowest survival rates, African Americans (AAs) are underrepresented in cancer research [15]. This underscores the need for AAs to participate more in cancer research to improve prevention and control.

The incidence of leukemia among U.S. Asians and foreign‐born Asian Americans is similar, suggesting a reduced risk relative to white populations [16]. Asians had the lowest rates of pediatric blood cancer, according to studies conducted in Europe and the UK, though the exact causes remain unknown [17]. Regardless of where they were born, Asians generally have the lowest prevalence of blood cancer; however, Asians born in the United States have slightly higher rates than Asians born outside [18].

According to projections, the United States' cancer rates will rise by 45% in the next 20 years, which could exacerbate the disparities in blood cancer among minorities, such as Asians and Pacific Islanders [19]. Socioeconomic factors contribute to these inequities, such as Pacific Islanders' lower insurance coverage. Delays in diagnosis, increased mortality, and decreased involvement in cancer research resulted from the increase in the percentage of people without insurance from 14.2% to 15.9% between 2000 and 2005 [19].

### 1.8 CAR‐T Cell Therapy

Cancer has emerged as a significant worldwide health threat, with a sharp rise in case incidence in recent years. Despite advancements in traditional treatment, managing those therapies remains complicated [20]. To decrease severe side effects, increase effectiveness, and raise survival rates, new treatments are required. Immunotherapy, a recent advancement, has shown significant promise. It uses immunological components, including cytokines, immune cells, and antibodies, to reduce cancer cells and minimize cytotoxic effects that damage healthy cells [13, 21].

Immunotherapy, including CAR‐T cell therapy, involves modified autologous T cells to specifically target and eliminate cancer cells while retaining healthy tissue. CARs improve T cells' capacity to recognize tumor antigens, which in turn triggers an immunological response [22].

Prior to reinfusion, a patient's T cells are extracted and modified to target cancer cells as part of CAR‐T therapy. To eliminate cancer, these cells identify tumor antigens and initiate cytotoxic reactions [13]. CAR‐T cell therapy selectively targets cancer cells, minimizing damage to healthy tissues, in contrast to chemotherapy, which may damage organs and have severe side effects [23].

The U.S. FDA approved two CAR‐T cell therapies for hematological malignancies, namely acute lymphoblastic leukemia and large B‐cell lymphomas. CAR‐T therapy has improved vastly over the last year, resulting in better clinical outcomes and longer remission periods [24].

### 1.9 Racial and Socioeconomic Disparities and CAR‐T Cell Therapy

Racial, ethnic, and socioeconomic disparities restrict the use of CAR‐T therapy, which offers hope to patients with blood cancer. Only 1% of African Americans and 5.4% of Hispanics participate in CAR‐T admissions, demonstrating the underrepresentation of minority groups in clinical trials [25]. Hispanic and Black patients with B‐cell lymphoma, multiple myeloma, and acute lymphoid leukemia are also impacted by barriers such as limited access, underrepresentation in trials, and language [26].

For low‐income minorities, transportation and related expenses create significant barriers because therapy requires regular visits to specialized clinics. Food, accommodation, and therapy management costs which are sometimes not covered by insurance, increase financial strain and increase the risk of relapses and adverse outcomes [27].

Improving healthcare access, particularly for CAR‐T cell therapy in blood cancers, requires addressing racial and socioeconomic disparities. These differences frequently result in lower health outcomes for socially disadvantaged communities. Future disparities can be avoided by identifying and addressing the underlying causes [28]. Racial and socioeconomic barriers still restrict access to CAR‐T therapy, which reduces the benefits for many patients despite medical improvements [5].

Resources have been stressed due to the increased demand for CAR‐T therapy, which has complicated patient selection and caused bias issues. For underprivileged populations, access and results could be increased by creating more medical centres and setting equitable frameworks in place [29]. This scoping review aims to map and synthesize existing evidence on racial and socioeconomic disparities in access to CAR–T cell therapy, an area critically underexplored despite its life‐saving potential.

## 2 Methodology

### 2.1 Guidelines Followed

This scoping review was conducted in accordance with established methodological frameworks, including the PRISMA‐ScR (Preferred Reporting Items for Systematic Reviews and Meta‐Analyses Extension for Scoping Reviews) guidelines (Table A1), to ensure transparency and reproducibility. The main research question was developed to examine the contemporary issue of socioeconomic and racial disparities in the accessibility of CAR‐T cell therapy for blood cancers. This question was designed to help focus the research and lead future studies into the causes, impacts, and possible solutions.

### 2.2 Eligibility Criteria

#### 2.2.1 Inclusion Criteria

To ensure the inclusion of high‐quality, peer‐reviewed evidence, the inclusion criteria focused on English‐language literature published since 2000 including high‐quality editorials and commentaries published in academic journals and gray literature that specifically addressed racial and socioeconomic disparities in access to CAR‐T cell therapy for blood cancers.

#### 2.2.2 Exclusion Criteria

The purpose of the exclusion criteria was to filter out research that was low‐quality, non‐peer reviewed or irrelevant. Studies on unrelated medical conditions or treatments have been excluded to keep the focus on CAR‐T cell therapy for blood cancers, and articles that were not published in English were excluded to ensure consistency in the study.

### 2.3 Data Sources

Data sources included widely used databases such as PubMed and Google Scholar, as well as gray literature and credible organization websites. The use of multiple sources facilitated comprehensive literature coverage, allowing for a thorough examination of racial and socioeconomic disparities in CAR‐T cell therapy accessibility.

### 2.4 Search Strategy

A systematic search strategy was applied to recognize relevant literature across multiple databases. Keywords and MeSH terms such as “health disparities,” “health equity,” “CAR‐T cell therapy,” and “blood cancers” were used to involve a wide range of appropriate studies. Boolean operators like “AND” “OR” and “NOT” enhanced search key strings for specificity. Examples included “health disparities” AND “CAR‐T cell therapy,” “health equity” AND “blood cancers,” and “CAR‐T cell therapy” NOT “solid tumors.” The search strategy was adapted to each database and documented to ensure reproducibility and transparency. The complete search strategy and corresponding outcomes are presented in Table A2.

### 2.5 PRISMA Flow Chart

**FIGURE 1:**
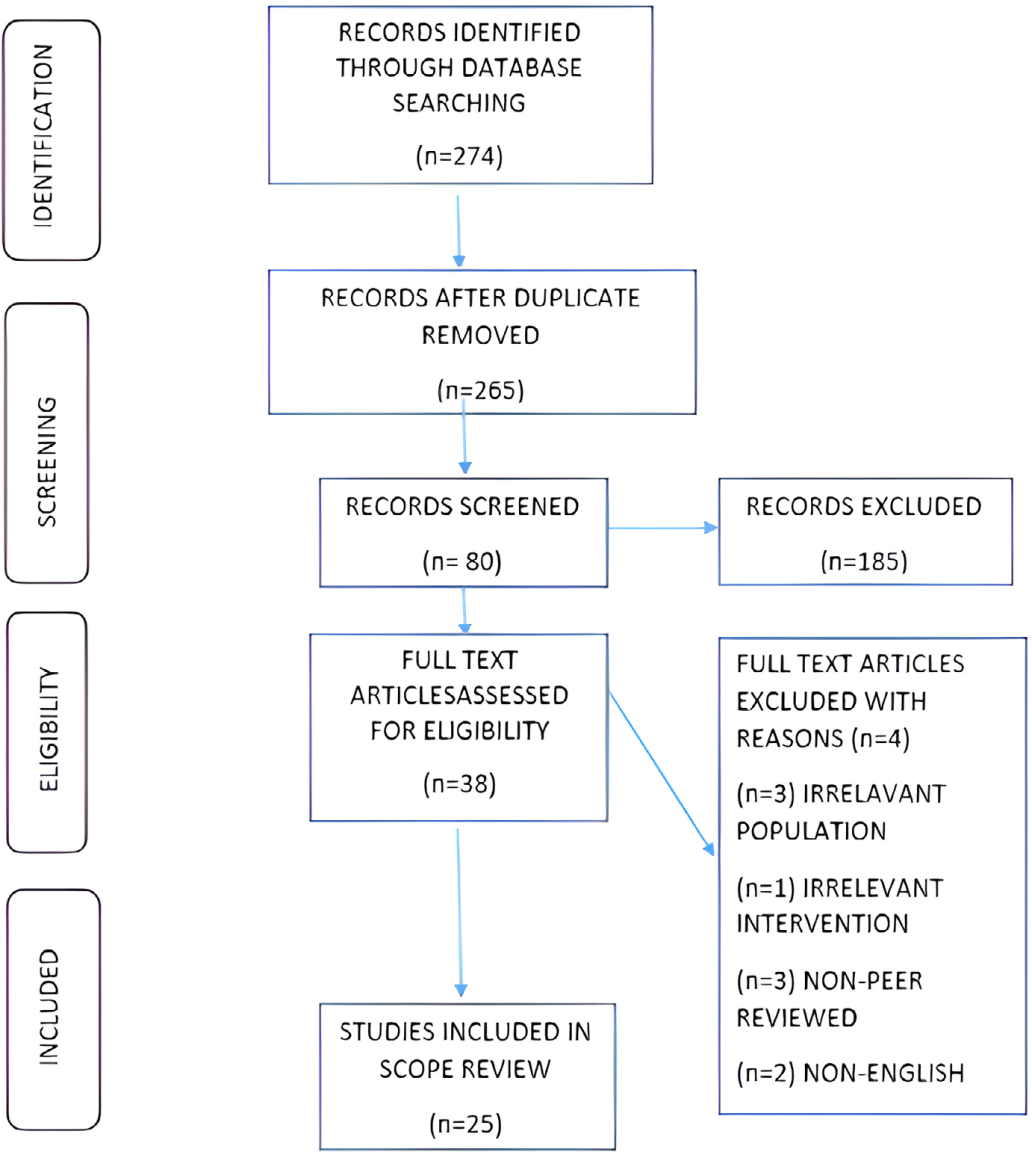
PRISMA flow chart: PRISMA flow diagram illustrating the study selection process for the scoping review. This flow diagram outlines the identification, screening, eligibility, and inclusion phases of the scoping review. A total of 274 records were initially identified through database searching. After removing duplicates, 265 records remained. Of these, 80 were screened, and 185 were excluded based on titles and abstracts. Full texts of 38 articles were assessed for eligibility, with 4 excluded for reasons including irrelevant population (*n* = 3), irrelevant intervention (*n* = 1), non‐peer‐reviewed sources (*n* = 3), and non‐English language (*n* = 2). Ultimately, 25 studies were included in the final review.

The PRISMA flow chart visualizes the systematic study selection process, which includes study identification, screening, eligibility, and inclusion (Figure 1). This chart increased transparency and repeatability by ensuring that all relevant research was documented.

### 2.6 Screening Process

Titles and abstracts retrieved from database searches were screened against the predefined inclusion criteria. Full‐text articles of potentially relevant studies were then assessed for eligibility. No formal quality appraisal tool was used; however, a standardized data extraction form was employed to ensure consistency in capturing key study characteristics and emerging themes throughout the review process.

### 2.7 Quality Appraisal

A basic quality appraisal was carried out to assess the credibility and relevance of the included studies. Two simple tools were developed to correspond with different types of literature. For reviews, book chapters, editorials, and commentaries, the appraisal looked at aspects such as whether the purpose was clear, the author had relevant expertise, the content was supported by evidence, and how credible and useful the source was. For empirical studies including descriptive, analytical, and experimental studies, the appraisal focused on whether the study had clear inclusion criteria, an appropriate design, valid measurements, adequate sample size, control of confounding factors, and appropriate data analysis. Each item was scored as 1 (yes) or 0 (no), and the total score was used to rate the overall quality as high (5–6), moderate (3–4), or low (below 3). While no formal critical appraisal tool was used, this basic approach helped maintain consistency and transparency in evaluating the quality of the diverse sources included in the review. The results of the quality appraisal are presented in Tables A3, A4.

### 2.8 Data Synthesis and Extraction

Data synthesis involved charting and grouping extracted data to identify repeated themes related to disparities in access to CAR‐T cell therapy. Studies were grouped thematically, with primary themes including racial, financial, educational, and broader socioeconomic disparities. Additional contextual factors included healthcare system differences, insurance coverage, geographic location, and health literacy.

### 2.9 Analysis

A thematic content analysis approach was applied to explore these themes in depth. This process aimed to highlight disparities across ethnic and socioeconomic groups, identify contributing factors, and assess their implications for treatment access, outcomes, and patient well‐being.

### 2.10 Ethical Considerations

As this study involved only secondary data from published sources, all data were anonymized, and no personal health information was accessed. Furthermore, all included articles were properly cited in accordance with academic integrity standards. The article selection and analysis were conducted objectively and without bias, based solely on relevance and quality rather than author identity, institutional affiliation, or publication source.

## 3 Results

The key findings from the thematic analysis are presented in Table 1. A detailed summary of each article, including key findings and relevance, is provided in Table A5.

**TABLE 1:** Key findings from the thematic analysis of selected articles.

Theme/findings	Citation	Study type	Setting
Negative impacts of socioeconomic and racial imparities in health	National Research Council (US) [3]	Book chapter	—
Education disparity in blood cancer related care in health sector	Jibu et al. [9] Montez and Cheng [30]	Cross sectional study Cross‐sectional study	Online questionnaire, Chennai, India USA
Minorities and worst health outcome and impact of education	Egede [6] Cutler and Lleras‐Muney [31]	Editorial Quantitative analysis	— USA
Influence of racial differences in health care	Williams and Sternthal [32]	Literature review	USA
Impact of financial disparity	Jaeggi et al. [4]	Observational analytical study	Tsimane, Bolivia
Financial and other socioeconomic disparities lead to unhealthy behavior.	Pampel et al. [8]	Literature review	USA
Gender disparity	Mirin [10]	Quantitative analysis	USA
Prevalence and insight of the severity of blood cancer	Siegel et al. [33]	Descriptive study	USA
Racial and ethnic minorities affect cancer largely	Kirtane and Lee [34]	Literature review	USA
Main blood cancer incidences in minorities	Kirtane and Lee [34]	Literature review	USA
Prevalence of blood cancer, diagnosis and prognosis	Howell et al. [12] Chu et al. [35]	Qualitative study Experimental study	UK China
Blood cancer prevalence in Hispanics, blacks and Asian/pacific islanders	Bencomo‐Alvarez et al. [14] Sheppard et al. [15] Pang et al. [16] Beale [19]	Commentary Descriptive study (*n* = 712) Cohort study (Asians and White) Commentary	USA USA USA USA
Importance of CAR‐T cell therapy to treat blood cancer	Morotti et al. [21] De Marco et al. [13] Sterner and Sterner [23] Zhang et al. [22]	Literature review Literature review Literature review Book chapter	USA — — USA
Disparities in CAR‐T cell therapy and future of the therapy	Ahmed et al. [25] Hall et al. [26] Yamamoto et al. [27] Bell et al. [29]	Cohort study Cohort study Analytical study Perspective study	USA USA Japan, USA USA

Articles from scientific databases such as PubMed, ScienceDirect and Nature were obtained to answer the knowledge gaps related to the research question of this report. After thorough investigation a total of 25 articles were selected to address the research aim and question. The duplicated and non‐English articles were excluded. Full text articles published since 2000 were selected. The selected articles and their findings, as presented in Table 1, were thematically categorized to address the key issues related to disparities in CAR‐T cell therapy access.

Several repeated factors contributing to health disparities were identified in the reviewed literature. Socioeconomic and racial disparities were regularly reported as major barriers to accessing CAR‐T cell therapy. Educational, financial, and gender‐related inequalities were also shown to intensify the gap in healthcare provision, especially among marginalized and minority populations. Ethnic and racial differences are contributing to health disparities in every country. Throughout the study, minority groups were identified and analyzed to understand the prevalent health disparities these minority groups are experiencing especially in the context of blood cancers.

The literature also highlighted the impact of disparities on treatment outcomes and the overall well‐being of individuals with blood cancers. This evidence is supported by several articles that outlined how disparities in CAR‐T cell therapy affect treatment outcomes and the overall patient experience. The socioeconomic disparities contributed by education, social status and financial disparities were shown to prevent patients from receiving the full benefit of these advanced treatments. Lack of knowledge of the CAR‐T cell treatment, financial inability to receive the treatment and racial or ethnic background are identified in the study as major health disparities in CAR‐T cell therapy access.

Several of the reviewed articles also proposed potential strategies and suggestions to minimize existing disparities in CAR‐T cell therapy. Minorities have low socioeconomic status, and it is negatively affecting them from receiving proper care for blood cancers. Therefore, policies that permit equitable access to each and every individual into care regardless of their racial and socioeconomic status need to be established. The individuals need to be granted accessible and affordable health care insurance to receive proper medication. The government also has to consider the families of the affected individuals and needs to prevent the economical, physical and psychological burden on them. Therefore, this study suggests the renewal and modification of existing policies and establishing newer policies that allow equitable access to CAR‐T cell therapy regardless of patient racial and socioeconomic status.

## 4 Discussion

### 4.1 Main Findings of This Study

CAR‐T cell therapy is an important cancer treatment, but health disparities limit access for underprivileged populations [36]. This article examines recent studies on racial and socioeconomic barriers to CAR‐T therapy. Minorities, particularly African Americans (AAs), are underrepresented in clinical trials, which limits obtaining the maximum health benefit from CAR‐T therapy. This gap highlights the need for greater minority involvement in trials to enhance health outcomes and improve the generalizability of clinical findings [4].

### 4.2 What Is Already Known on This Topic

Socioeconomic disparities restrict access to quality care. Financial, gender, and educational inequalities all contribute to these disparities. Low‐income populations have worse health outcomes because they cannot afford reliable medical care, whereas higher‐income populations have better access and outcomes [4]. Evidence shows that healthcare outcomes and access are impacted by one's position in the socioeconomic hierarchy, which defines one's relative access to resources [37].

Families may find it difficult to pay for CAR‐T cell therapy alongside other costs like food, accommodation, and travel, which could limit their ability to receive therapy. Relapses decrease survival rates, and worsened situations can result from this, which influences public health [27].

### 4.3 What This Study Adds

High mortality and low survival rates are indicators of insufficient healthcare, which has an impact on national politics and economics. These issues should be addressed in future initiatives, and collaboration will be necessary to discover effective solutions. In addition to ensuring that CAR‐T cell therapy treatments are cost‐effective, decision‐makers should advocate for insurance coverage of all associated expenses. To ensure equitable access to CAR‐T cell therapy, clinical practitioners need to develop strategies for equitable resource distribution that include eligible patients irrespective of race and socioeconomic backgrounds.

### 4.4 Limitations of This Study

This study has certain limitations. The exclusion of non‐English language studies may have omitted relevant international research. Additionally, reliance on published literature may introduce publication bias, as studies with statistically significant results are more likely to be published which were overcome by including gray literature.

Another limitation is that most of the included studies were conducted in the United States, a high‐income country according to the World Bank classification [38]. The significance of this is that even in a well‐resourced healthcare system, substantial socioeconomic and racial disparities in access to CAR‐T cell therapy persist. This highlights the likelihood that such disparities may be even more severe in low‐ and middle‐income countries (LMICs), where healthcare infrastructure, funding, and equitable access are more limited. Therefore, although the findings may not fully represent global contexts, they highlight universal challenges that are likely to exist and possibly be more severe, outside high‐income settings. This limitation may affect the generalizability of the findings and should be considered when interpreting the results.

The absence of data on African American involvement in CAR‐T cell therapy research is one of the study's limitations. However, it still provides important information on this minority group's access to CAR‐T cell therapy. Increasing AAs' engagement in CAR‐T cell therapy research must be one of the primary focuses of future studies, as it is essential for improving the generalizability of findings and ensuring equitable access to treatment [27].

## 5 Conclusion and Recommendations

This scoping review pinpoints important racial and socioeconomic inequities in access to CAR‐T cell therapy for patients with hematologic malignancies. These disparities limit timely diagnosis, clinical trial enrolment, and treatment access, especially among underprivileged groups.

The findings underscore the need for targeted policy reforms and strategic investments in equitable healthcare infrastructure. For example, studies [15] and [26] highlight the importance of establishing hospital‐based referral systems and community outreach programs to improve clinical trial participation among minority groups. Similarly, the study [25] emphasizes enrolling more underrepresented populations in CAR‐T trials to address racial and socioeconomic inequities and to ensure equitable access to CAR‐T therapy.

Future research should focus on inclusive recruitment strategies, socioeconomic considerations in healthcare delivery, and improved health literacy related to blood cancers, as noted by researchers [9] and [11]. Government‐led efforts, intersectoral collaboration, and the development of ethical frameworks for resource allocation as highlighted by the study [29] will be essential in ensuring that CAR‐T and other advanced therapies are accessible to all, regardless of race, ethnicity, or socioeconomic status.

## Author Contributions

**Hasini Warnakulasuriya:** conceptualization (lead), methodology (lead), writing – original draft (lead). **Ritika Tiwari:** supervision (lead), validation (lead), writing – review and editing (lead).

## Funding

The authors have nothing to report.

## Ethics Statement

The authors have nothing to report.

## Conflicts of Interest

The authors declare no conflicts of interest.

## Data Availability

No datasets were used or generated for this research article.

## Appendix 1 PRISMA Checklist

**TABLE A1:** Preferred Reporting Items for Systematic Reviews and Meta‐Analyses Extension for Scoping Reviews (PRISMA‐ScR) checklist.

Section	Item	PRISMA‐ScR checklist item	Reported on page #
Title			
Title	1	Identify the report as a scoping review.	1
Abstract			
Structured summary	2	Provide a structured summary that includes (as applicable): background, objectives, eligibility criteria, sources of evidence, charting methods, results, and conclusions that relate to the review questions and objectives.	2
Introduction			
Rationale	3	Describe the rationale for the review in the context of what is already known. Explain why the review questions/objectives lend themselves to a scoping review approach.	3, 4, 5, 6
Objectives	4	Provide an explicit statement of the questions and objectives being addressed with reference to their key elements (e.g., population or participants, concepts, and context) or other relevant key elements used to conceptualize the review questions and/or objectives.	7
Methods			
Protocol and registration	5	Indicate whether a review protocol exists; state if and where it can be accessed (e.g., a Web address); and if available, provide registration information, including the registration number.	N/A
Eligibility criteria	6	Specify characteristics of the sources of evidence used as eligibility criteria (e.g., years considered, language, and publication status), and provide a rationale.	8
Information sources^a^	7	Describe all information sources in the search (e.g., databases with dates of coverage and contact with authors to identify additional sources), as well as the date the most recent search was executed.	8
Search	8	Present the full electronic search strategy for at least 1 database, including any limits used, such that it could be repeated.	27
Selection of sources of evidence^b^	9	State the process for selecting sources of evidence (i.e., screening and eligibility) included in the scoping review.	10, 11
Data charting process^c^	10	Describe the methods of charting data from the included sources of evidence (e.g., calibrated forms or forms that have been tested by the team before their use, and whether data charting was done independently or in duplicate) and any processes for obtaining and confirming data from investigators.	11
Data items	11	List and define all variables for which data were sought and any assumptions and simplifications made.	11
Critical appraisal of individual sources of evidence^d^	12	If done, provide a rationale for conducting a critical appraisal of included sources of evidence; describe the methods used and how this information was used in any data synthesis (if appropriate).	11
Synthesis of results	13	Describe the methods of handling and summarizing the data that were charted.	11
Results			
Selection of sources of evidence	14	Give numbers of sources of evidence screened, assessed for eligibility, and included in the review, with reasons for exclusions at each stage, ideally using a flow diagram.	15
Characteristics of sources of evidence	15	For each source of evidence, present characteristics for which data were charted and provide the citations.	13, 14, 15
Critical appraisal within sources of evidence	16	If done, present data on critical appraisal of included sources of evidence (see item 12).	28, 29, 30
Results of individual sources of evidence	17	For each included source of evidence, present the relevant data that were charted that relate to the review questions and objectives.	13, 14
Synthesis of results	18	Summarize and/or present the charting results as they relate to the review questions and objectives.	16
Discussion			
Summary of evidence	19	Summarize the main results (including an overview of concepts, themes, and types of evidence available), link to the review questions and objectives, and consider the relevance to key groups.	17
Limitations	20	Discuss the limitations of the scoping review process.	18
Conclusions	21	Provide a general interpretation of the results with respect to the review questions and objectives, as well as potential implications and/or next steps.	19
Funding			
Funding	22	Describe sources of funding for the included sources of evidence, as well as sources of funding for the scoping review. Describe the role of the funders of the scoping review.	24

Abbreviations: JBI, Joanna Briggs Institute; PRISMA‐ScR, Preferred Reporting Items for Systematic reviews and Meta‐Analyses extension for Scoping Reviews.

^a^ Where *sources of evidence* (see second footnote) are compiled from, such as bibliographic databases, social media platforms, and websites.

^b^ A more inclusive/heterogeneous term used to account for the different types of evidence or data sources (e.g., quantitative and/or qualitative research, expert opinion, and policy documents) that may be eligible in a scoping review as opposed to only studies. This is not to be confused with *information sources* (see first footnote).

^c^ The frameworks by Arksey and O'Malley [39] and Levac and colleagues [40] and the JBI guidance [41, 42] refer to the process of data extraction in a scoping review as data charting.

^d^ The process of systematically examining research evidence to assess its validity, results, and relevance before using it to inform a decision. This term is used for items 12 and 19 instead of “risk of bias” (which is more applicable to systematic reviews of interventions) to include and acknowledge the various sources of evidence that may be used in a scoping review (e.g., quantitative and/or qualitative research, expert opinion, and policy document).

*Source:* Tricco et al. [43].

## Appendix 2 Search Strategy

**TABLE A2:** Search strategy adapted for each database with the outcome.

Search engine	Search key string	Number of results returned	Article identified
Google Scholar	(“Health disparities” OR “Health equity”) AND (“CAR‐T cell therapy” OR “Chimeric antigen receptor T‐cells”) AND (“Blood cancers” OR “Hematologic malignancies” OR Leukemia OR Lymphoma) NOT (“Solid tumors”)	240	27
	(“Health disparities”) AND (“CAR‐T cell therapy” OR “Chimeric antigen receptor T‐cells”)	1110	221
	(“Health disparities”) AND (“CAR‐T cell therapy” OR “Chimeric antigen receptor T‐cells”) AND (“Blood cancers”)	1030	18
PubMed	(“Health disparities” OR “Health equity”) AND (“CAR‐T cell therapy” OR “Chimeric antigen receptor T‐cells”) AND (“Blood cancers” OR “Hematologic malignancies” OR Leukemia OR Lymphoma) NOT (“Solid tumors”)	3	3
	(“Health disparities”) AND (“CAR‐T cell therapy” OR “Chimeric antigen receptor T‐cells”)	1	1
	(“Health disparities”) AND (“CAR‐T cell therapy” OR “Chimeric antigen receptor T‐cells”) AND (“Blood cancers”)	0	0
Gray Literature and Websites	“Health disparities” AND “CAR‐T cell therapy”	9	4

## Appendix 3 Results of Quality Appraisal

**TABLE A3:** Quality appraisal for empirical studies.

Study (author, year)	Design	Clear inclusion criteria	Appropriate design	Valid measurements	Confounders controlled	Data analysis appropriate	Sample size adequate	Total score	Overall quality (high/mod/low)
Pang et al. (2002)	Cohort study	1	1	1	1	1	1	6	High
Sheppard et al. (2021)	Descriptive study	1	1	1	0	1	1	5	High
Bell et al. (2023)	Perspective study	1	1	0	0	1	0	3	Moderate
Ahmed et al. (2022)	Cohort Study	1	1	1	1	1	1	6	High
Chu et al. (2020)	Experimental study	1	1	1	0	1	0	4	Moderate
Siegel et al. (2021)	Descriptive study	1	1	1	1	1	1	6	High
Jaeggi et al. (2021)	Observational analytical study	1	1	1	1	1	1	6	High
Montez and Cheng (2022)	Cross sectional study	1	1	1	1	1	1	6	High
Mirin (2021)	Quantitative analysis	1	1	1	0	1	1	5	High
Hall et al. (2023)	Cohort study	1	1	1	1	1	1	6	High
Yamamoto et al. (2024)	Analytical Study	1	1	1	1	1	1	6	High
Howell et al. (2022)	Qualitative study	1	1	1	0	1	1	5	High
Cutler and Lleras‐Muney (2008)	Quantitative analysis	1	1	1	1	1	1	6	High
Jibu et al. (2022)	Cross sectional study	1	1	1	0	1	0	4	Moderate

**TABLE A4:** Quality appraisal for non‐empirical studies.

Study (author, year)	Design	Clear purpose or main argument	Author expertise	Evidence or reference support	Relevance	Contribution to discourse	Source credibility	Total score	Overall quality (high/mod/low)	
Bencomo‐Alvarez et al. (2021)	Commentary	1	1	1	1	1	1	6	High	
Beale (2010)	Commentary	1	1	1	1	1	1	6	High	
Egede (2006)	Editorial	1	1	1	1	1	1	6	High	
Sterner and Sterner (2021)	Literature review	1	1	1	1	1	1	6	High	
Morotti et al. (2021)	Literature review	1	1	1	1	1	1	6	High	
Kirtane and Lee (2017)	Literature review	1	1	1	1	1	1	6	High	
De Marco et al. (2023)	Literature review	1	1	1	1	1	1	6	High	
Pampel et al. (2010)	Literature review	1	1	1	1	1	1	6	High	
Williams and Sternthal (2010)	Literature review	1	1	1	1	1	1	6	High	
Zhang et al. (2022)	Book chapter	1	1	1	1	1	1	6	High	
National Research Council (US) (2004)	Book chapter	1	1	1	1	1	1	6	High	

## Appendix 4 Results

**TABLE A5:** Detailed summary of reviewed articles.

Title of the article	Citation	Setting/conclusion	Future recommendation
Race/ethnicity, socioeconomic status, and health	National Research Council (US) [3]	Racial/ethnic differences in morbidity and mortality tied to socioeconomic status in USA.	Collection of more details regarding the health disparities in minor groups. Incorporation of theoretical ideas into practical. Enhancing the national data related to racial and ethnic differences.
Awareness of hematological malignancies among college students	Jibu et al. [9]	Evaluation of knowledge regarding blood cancer among participants. Majority 68.9% were aware, and rest is not aware.	Quality improvement program to reduce knowledge disparity and improve standard of care.
Educational disparities in adult health across U.S. states: larger disparities reflect economic factors	Montez and Cheng [30]	Data collected from behavioral risk factor surveillance system during 2011–2018. Adults aged between 25 and 64. Estimation of economic, behavioral, family and healthcare mechanisms.	Important reductions in educational disparities in health may improve overall health.
Race, ethnicity, culture, and disparities in health care	Egede [6]	Evaluation of the factors that are driven by variations of health by using 3 studies.	Need more funding research to establish the validity and reliability of constructing instruments across racial and ethnic and cultural groups.
Understanding differences in health behaviors by education	Cutler and Lleras‐Muney [31]	Understanding the possible explanation for education and health behaviors.	More research needs to understand the possibility of income health insurance and family background can account for educational and health behaviors.
Understanding racial‐ethnic disparities in health: sociological contributions	Williams and Sternthal [32]	Analysis of social construct of race and ethnicities affect healthcare disparities.	Sociological notions on racial disparities in health have important implications for the improvement of health care equities.
Do wealth and inequality associate with health in a small‐scale subsistence society?	Jaeggi et al. [4]	Participants were used to assessing the relative socio‐economic position that affects health (*n* = 871).	Wealth and inequality are associated with several health outcomes.
Socioeconomic disparities in health behaviors	Pampel et al. [8]	Unhealthy lifestyle patterns among low socioeconomic status may lead lower health.	Evaluation of effects of more lifestyle patterns needs more study designs. Adopting innovative health behaviors can minimize unhealthy lifestyle patterns.
Gender disparity in the funding of diseases by the U.S. national institutes of health	Mirin [10]	Understanding the gender disparity in health care.	The authorities allocate more resources on men compared to women. Policy modification that allows more resource allocations for the women is needed.
Cancer statistics, 2021	Siegel et al. [33]	Estimation of new cancer cases and population‐based deaths data in all cancer forms including blood cancer.	Reduction of cancers gained by improvement of management of the disease and using the innovative treatment options.
Racial and ethnic disparities in hematologic malignancies	Kirtane and Lee [34]	Identifying differences in care that may lead to racial and ethnic disparities.	Future public health research needs to increase the participation of minor groups in clinical trials.
incurable but treatable: understanding, uncertainty and impact in chronic blood cancers—a qualitative study from the UK's hematological malignancy research network	Howell et al. [12]	The data collected to understand the awareness of patients about the disease. Evaluation of patients' education level on blood cancers treatability and curability.	Many patients lack knowledge about hematological malignancies. More education programs are needed to reduce the knowledge gaps in blood cancers.
Blood cancer diagnosis using ensemble learning based on a random subspace method in laser‐induced breakdown spectroscopy	Chu et al. [35]	Overcome challenges in diagnosing blood cancer using a recent innovative method.	Identifying blood cancer in early stages improves the survival rates of the patients.
Blood cancer health disparities in the United States hispanic population	Bencomo‐Alvarez et al. [14]	Existing knowledge about the health disparities in minor groups who are affected by blood cancers in USA.	Conclusion‐ younger Hispanics patients have higher incidents rates in blood cancers and worse prognosis. Better strategies to understand underlining factors may reduce or eliminate the disparity.
Recruitment of african americans into cancer clinical research: strategies and outcomes	Sheppard et al. [15]	Understand the factors affecting the racial disparities in cancer.	Healthcare Stakeholders need to combine diverse groups to engage larger number of minorities in cancer studies to improve morbidity and mortality.
Incidence of leukemia in Asian migrants to the United States and their descendants	Pang et al. [16]	To understand the blood cancer incidence rates among Asian American immigrants and their descendants with white population.	Regardless of birthplace Asian American possess low risk for blood cancer compared to US whites.
Identifying the causes of cancer health disparities: biologic and non‐biologic determinants	Beale [19]	Analysis of the causes of health disparities in cancer.	Interplay between biological and non‐biological factors may lead to health disparities in terms of prevalence, morbidity and mortality in certain population.
Promises and challenges of adoptive T‐cell therapies for solid tumors	Morotti et al. [21]	Evaluation of advancement of CAR‐T cell therapy in terms of recognition of cancer cells and elimination of tumors.	CAR‐T cell therapy is a credible therapeutic option for blood cancer. CAR‐T cell therapy may have enormous potential to improve clinical outcomes of cancers.
CAR‐T cell therapy: a versatile living drug	De Marco et al. [13]	Evaluation of current development and clinical outcomes of CAR‐T cell therapy.	Future development of CAR‐T cell therapy may decrease the disparities in cancer related incidents in health care.
CAR‐T cell therapy: current limitations and potential strategies	Sterner and Sterner [23]	Analyzing the current advancement and limitation of CAR‐T cell therapy.	Recommendations to improve anti‐tumor activity and other limitations in CAR‐T cell therapy for better clinical efficacy.
Chimeric antigen receptor (CAR)‐T cell therapy	Zhang et al. [22]	Clinical significance of CAR‐T cell therapy as an efficient treatment against cancers.	Possibility of improving better care with CAR‐T cell therapy in association with interprofessional health care team give best remarks in future
Socioeconomic and Racial Disparity in Chimeric Antigen Receptor T Cell Therapy Access	Ahmed et al. [25]	Efforts to address disparities exist in CAR‐T cell therapy and to ensure availability of life saving therapy to everyone.	Future strategies based on enrolling the more minorities in CAR‐T cell‐related clinical trials may resolve the inequity.
Cost‐effectiveness of anti‐BCMA chimeric antigen receptor T Cell therapy in relapsed/refractory multiple myeloma	Yamamoto et al. [27]	Cost effectiveness of CAR‐T cell therapy. Evaluation of equity of access to CAR‐T cell therapy.	Changes in certain settings in healthcare may influence the cost effectiveness of CAR‐T cell therapy.
Access to chimeric antigen receptor T cell clinical trials in underrepresented populations: a multicenter cohort study of pediatric and young adult acute lymphoblastic leukemia patients	Hall et al. [26]	Evaluation of feasibility of accessing the CAR‐T cell therapy to patients of low socioeconomic status or racial or ethnic minor groups.	Encouraging minorities in participating in clinical trials related to CAR‐T cell therapies. Establishing partnerships with health care settings, hospitals to encourage patient referral and patient access to CAR‐T cell clinical trials.
Mitigating inequity: ethically prioritizing patients to CAR T‐cell therapy	Bell et al. [29]	Managing the limited resources allocated for CAR‐T cell therapy for the eligible population.	Ethical framework needed to find and allocate the limited resources available in CAR‐T cell therapy. Fairness and equity in allocating the resources regardless of racial and socioeconomic disparities.
